# Hepatic DNA Methylation in Response to Early Stimulation of Microbiota with *Lactobacillus* Synbiotics in Broiler Chickens

**DOI:** 10.3390/genes11050579

**Published:** 2020-05-21

**Authors:** Aleksandra Dunislawska, Anna Slawinska, Maria Siwek

**Affiliations:** Department of Animal Biotechnology and Genetics, UTP University of Science and Technology, 85-796 Bydgoszcz, Poland; slawinska@utp.edu.pl (A.S.); siwek@utp.edu.pl (M.S.)

**Keywords:** gut–liver axis, gene silencing, prebiotic, probiotic

## Abstract

DNA methylation inhibits DNA transcription by the addition of methyl residues to cysteine within the CpG islands of gene promoters. The process of DNA methylation can be modulated by environmental factors such as intestinal microbiota. In poultry, the composition of the intestinal microbiota can be stimulated by in ovo delivery of synbiotics. The present study aims to determine the effect of *Lactobacillus* synbiotics delivered in ovo on the level of hepatic DNA methylation in broiler chickens. In ovo stimulation was performed on day 12 of egg incubation. Bioactive compounds delivered in ovo included (S1)—*Lactobacillus salivarius* with GOS and (S2)—*Lactobacillus plantarum* with RFO. Samples were collected from six individuals from each group on day 42 post-hatching. DNA methylation of five genes selected on the basis of the transcriptome data were analyzed using the qMSP method. Significant changes were observed in DNA methylation of genes in liver including *ANGPTL4* and *NR4A3*, after S2 delivery. The obtained results confirm that the downregulation of metabolic gene expression in the liver mediated by in ovo stimulation had epigenetic characteristics.

## 1. Introduction

DNA methylation generally is one of the epigenetic mechanisms that inhibits DNA transcription by the addition of methyl residues to cysteine within the CpG islands of gene promoters. DNA methylation mostly silences gene expression, which is defined as epigenetic modification of gene expression. The process of DNA methylation can be modulated by environmental factors such as nutrition [1], stress [2], climate and intestinal microbiota [3]. Crosstalk between microbiome and host metabolism often leads to epigenetic regulation of gene expression [4]. The intestinal microbiota affects the regulation of gene expression in intestinal epithelial cells, which is directly related to DNA methylation [3]. Commensal gut microorganisms can modify the methylation pattern of epithelial cells and thus can modulate cell function [5]. DNA methylation plays an important role in controlling homeostasis and intestinal differentiation [6]. Intestinal colonization by microorganisms affects epigenetic mechanisms such as acetylation and methylation of histones not only in the gut, but also in many host tissues such as liver [7]. As the main metabolic organ, the liver plays a key role in nutrient metabolism, fat digestion, blood protein synthesis and endocrine management. The liver also has unique immunological properties because of the presence of immune cell repertoire and the capacity to recruit and activate immune cells in response to gut-derived metabolic signals [8,9]. Liver anatomy allows for close interaction with the intestines through the biliary tract, port vein and systemic circulation [10]. Thus, the liver is a central organ that mediates interactions between the intestinal microbiota and host metabolism. A large amount of metabolites in peripheral blood are of bacterial origin; hence, there is strong influence of bacteria on the entire host organism, especially on the liver and pancreas [8].

In poultry, composition of the intestinal microbiota can be stimulated during embryo development by bioactive compounds such as prebiotics, probiotics and synbiotics. In ovo administration of bioactive compounds influences mRNA gene expression in the immune, intestinal and metabolic tissues [11]. In ovo administration of *Lactobacillus*-based synbiotics modulated gene expression in the liver. Molecular regulation of pathways associated with metabolism and immune response was demonstrated, especially after the administration of *Lactobacillus plantarum* with raffinose family oligosaccharides (RFO). The administration of this synbiotic significantly caused changes in the expression of 159 genes, while the synbiotic *Lactobacillus salivarius* with galacto-oligosaccharides (GOS) affected the expression of 48 genes in broiler chicken liver [11].

We hypothesize that there is epigenetic regulation of hepatic mRNA gene expression in broiler chickens stimulated in ovo with *Lactobacillus* synbiotics. The objective of the present study was to determine the methylation profile of several genes that were regulated by *Lactobacillus* synbiotics at the transcriptomic level. Thus, we aimed to demonstrate that in ovo stimulation of the chicken microbiome leads to epigenetic reprogramming of metabolic and immune functions of the liver.

## 2. Materials and Methods

### 2.1. Selection of Gene Panel

The panel of immune- and metabolic-related genes was selected on the basis of the microarray analysis of liver samples harvested from chickens stimulated in ovo with *Lactobacillus* synbiotics. The microarray procedure was based on the Chicken Gene 1.1 ST Array Strip (Affymetrix, Santa Clara, CA, USA). The transcriptomic data were published elsewhere [5]. The main criteria for gene selection were strong downregulation of gene expression and statistical significance (*p* < 0.05).

### 2.2. Experimental Setup

A total of 5850 Cobb500FF eggs were incubated in standard conditions. On day 12 of egg incubation, the eggs were randomly distributed into the following experimental groups: synbiotic 1 (S1)—*Lactobacillus salivarius* with GOS and synbiotic 2 (S2)—*Lactobacillus plantarum* with RFO. The control group (C) was mock injected with physiological saline. The experimental setup, hatching results and production parameters were described in Dunislawska et al. 2017 [12]. Six randomly selected individuals from each group (S1, S2 and C) were sacrificed on day 42 post-hatching and their liver was collected.

### 2.3. DNA Extraction

DNA isolation from the liver (*n* = 6) was performed using the phenol–chloroform protocol [13]. A piece of tissue was placed in 2.0 mL Eppendorf tube containing 500 µL of lysis buffer (1-M Tris-HCl, 0.5 M EDTA, pH 8) and proteinase K. The tissues were homogenized using the TissueRuptor homogenizer (Qiagen, GmbH, Hilden, Germany). After homogenization, the samples were vortexed and centrifuged (12,000× *g*, 10 min). The supernatant was transferred to a new tube. An equal volume of phenol-chloroform-isoamyl alcohol was added to the supernatant, mixed and centrifuged (13,000 rpm, 10 min, room temperature). The aqueous phase was transferred to a new tube without disturbing the protein interphase and again centrifuged (13,000 rpm, 10 min, 4 °C). The DNA precipitate was washed with 70% ethanol and left to dry. The dry precipitate was dissolved in TE buffer overnight in room temperature. DNA was subjected to qualitative (electrophoresis on 2% agarose gel) and quantitative (Scientific Nanodrop Products, Wilmington, DE, USA) assessments.

### 2.4. Real-Time Quantitative Methylation-Specific Polymerase Chain Reaction (*qMSP*)

The isolated DNA was subjected to methylation analysis using the Real-Time Quantitative Methylation-Specific Polymerase Chain Reaction (qMSP). The qMSP method is a methylation-specific quantitative PCR preceded by DNA bisulfite conversion. Bisulfite conversion was performed using the EpiJet Bisulfite Conversion Kit (Thermo Fisher Scientific, Valtham, MA, USA) according to the manufacturer’s instructions. During bisulfite conversion, unmethylated cytosine undergoes deamination and transforms into uracil. In contrast, 5-methylcytosine (which is a methylated cytosine) is resistant to bisulfite conversion. Thus, methylated and unmethylated qPCR products can be distinguished using two pairs of primers specific for methylated and unmethylated DNA. The primers for qMSP reactions were designed within the CpG islands of gene promoters. Primer design was facilitated by the MethPrimer tool [14]. Primers for selected genes in two variants were designed: methylated and unmethylated. Primers for qMSP were complementary to the gene promoter region and were designed using the following criteria: island size > 100, GC% > 50.0; and obs./exp > 0.60 [15]. DNA oligonucleotides were synthesized by Sigma-Aldrich (presented in Table 1). The qPCR analysis was performed in the LightCycler 480 (Roche Diagnostics, Risch-Rotkreuz, Switzerland) thermal cycler. The reaction mixture contained the Maxima SYBR Green qPCR Master Mix intercalating dye (Thermo Fisher Scientific, Valtham, MA, USA). The optimized melting temperature was 58 °C. After amplification, a melting curve was generated for each product (*n* = 6). The melting curve was obtained by a gradual increase in temperature up to 98 °C with continuous measurement of fluorescence. The relative level of DNA methylation [%] was calculated on the basis of the results of melting curves (read fluorescence level) for each individual according to the formula [16]: $\%\;methylation=100\times \left(\;\frac{M}{M+U}\right),$ where M—average fluorescence intensity of the methylated product and U—average fluorescence intensity of the unmethylated product. Statistical analysis was performed using Student’s *t*-test (*p* < 0.05).

## 3. Results

### 3.1. Gene Panel Selection and Primer Design

A panel of five genes (*ANGPTL4, NR4A3, CYR61, KLHL6* and *SYK*) was selected on the basis of the microarray data. The microarray results showed that the administration of S1 affected the expression of genes associated with metabolism and immune response, while the administration of S2 influenced the expression of genes related to metabolism and development of the organism. Three of the selected genes have metabolic functions (*ANGPTL4*, *NR4A3* and *CYR61*), while two genes (*SYK* and *KLHL6*) were associated with the immune system. According to the microarray data, the log-fold change value of *ANGPTL4* was −1.08 in S1 and −10.51 in S2. *CYR61* had a value of −4.5 after S1 delivery and −1.28 in the S2 group in the liver. The log-fold change of *NR4A3* was −5.24 in the S1 group but was 3.19 in the S2 group in the liver. *KLHL6* and *SYK* were not expressed in the liver but were expressed in immune tissue. The log-fold change of *KLHL6* and *SYK* was −2.15 and −1.72, respectively, in after S1 administration. The microarray data are presented in Table 2.

### 3.2. Gene-Specific Methylation in the Liver (qMSP Reaction)

**Figure 1:**
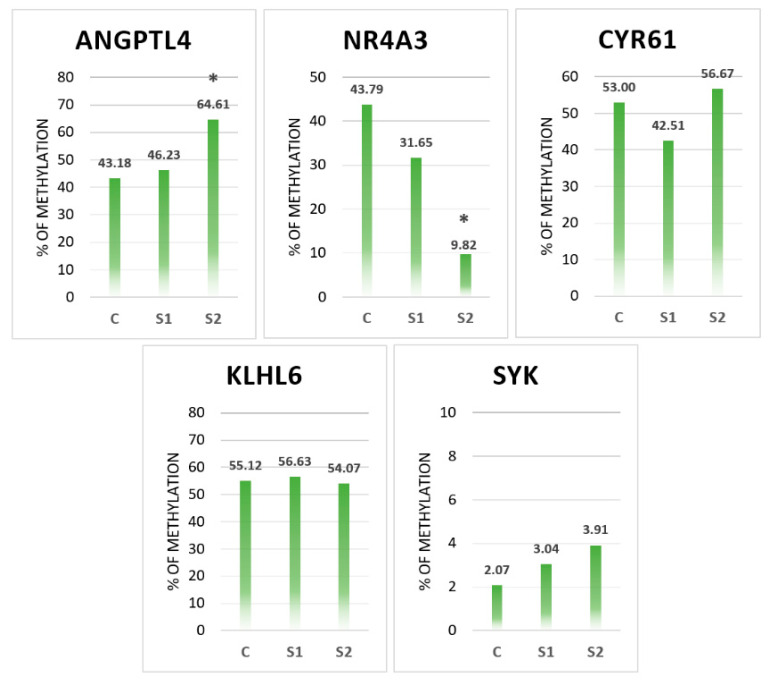
DNA methylation of the *ANGPTL4, NR4A3, CYR61, KLHL6* and *SYK* genes in the liver. X-axis—groups: C—control, S1—*Lactobacillus salivarius* with GOS, S2—*Lactobacillus plantarum* with RFO; Y-axis—percentage of methylation. * *p* < 0.05 (*n* = 6).

Figure 1 presents the relative level of DNA methylation for the analyzed genes. The methylation level of the metabolic genes *ANGPTL4* and *NR4A3* was significantly different in S2 compared to that in C (*p* < 0.05). An increase in methylation was detected for *ANGPTL4* in S1 (46.23%) and S2 (64.61%) compared to that in C (43.18%). The methylation level of *NR4A3* decreased in S1 (31.65%) and S2 (29.82%) compared to that in C (43.79%). The methylation level of the *CYR61* gene decreased after S1 administration (41.51%) and increased in the S2 group (56.67%) compared to that in C (53%) (*p* > 0.05).

The methylation level of the immune genes *KLHL6* and *SYK* did not change as compared to that in control.

## 4. Discussion

The present study aimed to determine the effect of the in ovo administration of *Lactobacillus* synbiotics on day 12 of egg incubation on the level of DNA methylation of several selected genes based on the transcriptome data in the liver tissue. Epigenetic DNA methylation affects gene activity, thereby providing a reliable molecular mechanism for a wide range of biological processes and diseases. As stated in the Introduction section, the liver was selected as an organ with metabolic and immunological functions. Therefore, in our opinion, liver tissue is a good choice for the first methylation analysis after the administration of bioactive substances in ovo on day 12 of egg incubation and microbial stimulation in broiler chickens. The in ovo technology applied on day 12 of egg incubation involves the administration of bioactive substances during embryonic development and thus stimulation of native intestinal microbiota before hatching [17]. The perinatal period is crucial for reprogramming the intestinal microbiota, thereby enabling the colonization of the gastrointestinal tract with beneficial bacteria before hatching [18]. The synbiotic administered to the air cell of the egg is a comprehensive solution. A small molecule of the prebiotic component can penetrate the subcutaneous membrane and enter the embryonic circulatory system. The probiotic becomes available during hatching when a chick leaves the shell, i.e., when the eggshell membrane is broken [17]. Indigenous microbiota plays a very important role in affecting epigenetic processes in the organism. The in ovo administration of bioactive substances is a potent method for stimulating embryonic microbiota that is likely to show persistent (epigenetic) changes in gene expression. The in ovo administration of synbiotics did not increase embryo mortality [12].

The in ovo delivery of S2 led to hypermethylation (decrease of 21%) of the *ANGPTL4* gene in the liver. Hypermethylation, i.e., an increased degree of methylation, causes silencing of gene expression. This is in agreement with the whole transcriptome analysis [11]. *ANTPTL4* expression was downregulated after the administration of synbiotics in the liver; especially, the expression was significantly decreased after the administration of S2 (10-fold decrease in expression compared to that in the control). This translates into the result of methylation of gene promoters. The *ANGPTL4* gene is involved in the pathways associated with the reduction of lipoprotein lipase (LPL) activity, triglyceride homeostasis and angiogenesis. It encodes a protein that regulates glucose homeostasis, lipid metabolism and insulin sensitivity. It was shown that *Lactobacillus* probiotic bacteria have an effect to lower body fat levels with higher *ANGTPL4* expression levels [19]. The *ANPTL4* gene is responsible for the inhibition of LPL, which leads to a decrease in fat storage [20]. Studies in mice have shown that the *ANGPTL4* gene and the intestinal microbiota control body weight [19]. The action of LPL also affects the release of a large amount of fatty acids from lipoproteins, which are taken up by tissues for energy production or storage [21]. In broiler chickens stimulated in ovo with *Lactobacillus* synbiotics in this experiment, the body weight of the experimental groups was not significantly different from that of the control group [12]. Meat quality analysis showed that the administration of S2 affected fatty acid profile and lipid content, which has a positive effect on the nutritional properties of poultry meat [22]. *Lactobacillus* synbiotics-mediated beneficial changes in the gene expression in the breast muscle of broiler chickens are associated with the improvement of muscle energy metabolism, glucose homeostasis and insulin sensitivity [23].

*NR4A3* methylation decreased after the administration of synbiotics. In particular, significant hypomethylation of *NR4A3* was observed after S2 administration. DNA hypomethylation refers to the loss of a methyl group in the 5-methylcytosine nucleotide. This causes a decrease in the percentage of methylated cytosines compared to that of unmethylated cytosines [24]. At the gene expression level, there was a decrease in expression after S1 administration, although the methylation level also decreased. Mossman and Scott [25] suggest that hypomethylation alone is often insufficient to reactivate the silenced genes. On the other hand, a significant decrease in the level of *NR4A3* methylation after S2 administration is strongly correlated with a significant increase in the *NR4A3* expression of this gene. *NR4A3* encodes a member of the steroid–thyroid–retinoid hormone receptor. It is a transcription activator that binds to regulatory elements in the promoter region. *NR4A3* plays an important role in the regulation of fatty acid use, muscle mass [26], cell proliferation and differentiation and apoptosis [27]. It can also promote food intake and body weight gain, as demonstrated in the mouse model [28]. The mRNA expression of this gene was significantly reduced in mildly obese mice [28]. In the current study, the gene expression of *NR4A3* was significantly positively regulated after S2 administration, which correlated with the low level of methylation. Assuming that there is an increase in methylation during gene silencing, a decrease in methylation would explain the increase in gene expression. However, body weight gain (BWG) and feed intake (FI) not differ significantly between the groups [12]. These changes in BWG and FI may be closely related to the substance delivered in ovo. It has been shown that the administration of *Lactococcus*-based synbiotics significantly increased BWG with unchanged feed conversion efficiency (FCE) [29] while the administration of prebiotics alone (RFO and GOS) increased BWG, FI and FCE [30]. References regarding the level of expression and methylation of the *NR4A3* gene in the liver, which could explain the observed changes in BGW and FCE related to prebiotic administration, are scarce and thus, this topic requires thorough research. On the basis of our research study, we conclude that this may be strongly associated with the delivered substance or its composition.

Methylation of gene promoters leads to the inhibition of transcription and consequently downregulation of mRNA expression. However, an experiment performed by Hu et al. [31] based on in ovo injection of betaine to the yolk membrane to determine the level of promoter methylation of selected genes in the liver proved that changes in methylation in response to substances are gene-specific. In addition, there was no analogous correlation between gene expression and methylation levels as detected in the current study. These authors also noted a mismatch between the promoter methylation level and the mRNA level. This may be due to the fact that many factors can influence the complex transcriptional regulation of gene expression. The methylation level of specific genes may respond differently than on the level of the global genomic DNA [32]. Differences in the effects of various substances on particular genes are also justified. DNA requires donors of methyl groups from the external environment such as food or supplementation. It has been shown that changes in the level of DNA methylation can be affected by the short-chain fatty acid butyrate, which is a product of prebiotic fermentation [33,34]. The regulation of the methylation process may depend on how the synbiotic acts upon administration in ovo. The tested synbiotics showed two different mechanisms of action. The first mechanism (described for S2) assumes that the synbiotic is synergistic for the host (prebiotic is less efficiently used by the delivered probiotic bacteria), thereby improving the balance of the intestinal microbiota while being available to other microorganisms. The second mechanism assumes that the components of the synbiotic are synergistic with respect to each other (S1) [12]. Further analysis is required to analyze the effect of individual components on DNA methylation.

The level of methylation of the immune-related genes *SYK* and *KLHL6* did not change after treatment with *Lactobacillus* synbiotics. *SYK* regulates several biologic processes, including innate and adaptive immunity and intermediate, while *KLHL6* encodes a protein involved in B-cell antigen receptor signaling and B-cell maturation at the embryonic center. In the microarray analysis, the level of expression of these genes changed in the immune tissue, while it remained unchanged in the liver relative to the control group injected with saline [11]. Thus, the level of DNA methylation is tissue-specific [35] and therefore, changes in methylation regulation in different tissues are justified. A high level of methylation is correlated with low or no transcription [36].

In conclusion, it was proved that the in ovo administration of synbiotics on day 12 of incubation of eggs affects the regulation of the level of methylation of genes associated with metabolism in the liver. Early stimulation of chicken intestinal microbiota has a strong effect on permanent changes in the DNA methylation profile. Epigenetic changes can be inherited by offspring, so that by administering bioactive substances, it is possible to stably modulate metabolism in chickens. It also seems reasonable to speculate that a well-matched bioactive substance delivered in ovo may support further grow and development of the adult chicken broiler.

## Figures and Tables

**Table 1:** Sequences of the primers designed for qMSP reaction by using the MethPrimer tool.

Gene		Primer Sequence	GC%	Amplicon Size	NCBI No.
**ANGPTL4**	M	F: TAATTTTAACGGGAAGTATTTTCGT R: CAACTTTAAAACTCTACCTCCAACG	56.00 60.00	156	769087
	U	F: TAATTTTAATGGGAAGTATTTTTGT R: ACTTTAAAACTCTACCTCCAACACA	56.00 60.00	154	
**NR4A3**	M	F: GGGAAAGGATAAAGTTTTTGTAGTC R: AAACTCAAACGTAACCCTAAACGTA	52.00 56.00	179	420996
	U	F: GGGAAAGGATAAAGTTTTTGTAGTTG R: AAACTCAAACATAACCCTAAACATA	53.85 56.00	179	
**CYR61**	M	F: TTTGGTTTTAGTGTTTAAAGACGT R: TTATATTTACCTTCAAAAAAACGTA	58.33 44.00	150	429089
	U	F: TTTTGGTTTTAGTGTTTAAAGATGT R: TATTTATATTTACCTTCAAAAAAACATA	56.00 42.86	154	
**KLHL6**	M	F: TTTTTTGGATAATGAGTGTTTAACG R: AAACACCAAAAAAAATCCCGTA	52.00 63.64	100	424762
	U	F: TTTTTGGATAATGAGTGTTTAATGA R: CTAAAACACCAAAAAAAATCCCATA	48.00 64.00	102	
**SYK**	M	F: TATTAGGCGTTTTCGGGAAC R: AAATTAATACATTTACTCGCCGCT	70.00 54.17	115	427272
	U	F: GTTTATTAGGTGTTTTTGGGAATGA R: CCAAATTAATACATTTACTCACCACT	68.00 57.69	120	

M—specific for methylated DNA; U—specific for unmethylated DNA.

**Table 2:** Gene expression in the liver after the in ovo administration of *Lactobacillus* synbiotics.

Gene	Pathway	S1		S2	
		Up/Down	FC	Up/Down	FC
*ANGPTL4*	Metabolic	−	−1.08	− −	−10.51
*NR4A3*		− −	−5.24	+ +	3.19
*CYR61*		− −	−4.5	−	−1.28
*KLHL6*	Immune	NC	1	NC	1
*SYK*		NC	1	NC	1

S1—*Lactobacillus salivarius* with GOS; S2—*Lactobacillus plantarum* with RFO; + upregulated; − downregulated; NC—no changes of gene expression. Calibrator assumes a value of 1. Based on microarray data published by Dunislawska et al. (2019) [11].
